# Gait parameter fitting and adaptive enhancement based on cerebral blood oxygen information

**DOI:** 10.3389/fnhum.2023.1205858

**Published:** 2023-07-24

**Authors:** Haozhe Ma, Chunguang Li, Yufei Zhu, Yaoxing Peng, Lining Sun

**Affiliations:** Key Laboratory of Robotics and System of Jiangsu, School of Mechanical and Electric Engineering, Soochow University, Suzhou, China

**Keywords:** functional near-infrared spectroscopy, cerebral oxygen, walk, motion parameter fitting, adaptive boosting

## Abstract

Accurate recognition of patients’ movement intentions and real-time adjustments are crucial in rehabilitation exoskeleton robots. However, some patients are unable to utilize electromyography (EMG) signals for this purpose due to poor or missing signals in their lower limbs. In order to address this issue, we propose a novel method that fits gait parameters using cerebral blood oxygen signals. Two types of walking experiments were conducted to collect brain blood oxygen signals and gait parameters from volunteers. Time domain, frequency domain, and spatial domain features were extracted from brain hemoglobin. The AutoEncoder-Decoder method is used for feature dimension reduction. A regression model based on the long short-term memory (LSTM) model was established to fit the gait parameters and perform incremental learning for new individual data. Cross-validation was performed on the model to enhance individual adaptivity and reduce the need for individual pre-training. The coefficient of determination (R2) for the gait parameter fit was 71.544%, with a mean square error (RMSE) of less than 3.321%. Following adaptive enhancement, the coefficient of R2 increased by 6.985%, while the RMSE decreased by 0.303%. These preliminary results indicate the feasibility of fitting gait parameters using cerebral blood oxygen information. Our research offers a new perspective on assisted locomotion control for patients who lack effective myoelectricity, thereby expanding the clinical application of rehabilitation exoskeleton robots. This work establishes a foundation for promoting the application of Brain-Computer Interface (BCI) technology in the field of sports rehabilitation.

## 1. Introduction

The aging population has become increasingly significant in recent years (Cohen, 2003). Elderly individuals often experience motor dysfunction due to reduced physical fitness (Carr, 2013). Furthermore, the number of individuals experiencing motor impairments due to traffic accidents, work injuries, and diseases is steadily rising. Lower-limb exoskeleton robots that facilitate patients’ lower-limb movements have the potential to enhance motor neuroplasticity and significantly improve rehabilitation outcomes (Zheng et al., 2019; Moucheboeuf et al., 2020). Ensuring a favorable human-robot interaction between the lower limb rehabilitation exoskeleton robot and the human body is crucial for ensuring safety, comfort, and the effectiveness of rehabilitation (Long et al., 2015). The BLEEX exoskeleton (Kazerooni et al., 2005; Zoss et al., 2005, 2006) detects the wearer’s gait by using plantar pressure, angle, and speed sensors for exoskeleton control. The eLEGS (Campbell, 2010; Strausser and Kazerooni, 2011) exoskeleton mainly utilizes sensors in the sensing crutches and lower limb skeleton to determine the human motion intention for human-robot coordination. However, traditional physical sensors can only detect the signal after the wearer’s movement, which makes it difficult to achieve precise control and real-time adjustment of the exoskeleton.

For the first time, the HAL exoskeleton (Hayashi et al., 2005; Kawamoto and Sankai, 2005) used electromyography (EMG) sensors to collect EMG signals, combined with knee torque and attitude sensors to identify human gait. EMG is a bioelectrical signal generated based on the action unit action potential sequence of muscle fibers during muscle activity, which can objectively reflect muscle activity information. Li et al. (2015) fitted the joint angle of the knee and hip based on EMG and used the least-squares support vector machine to establish a regression model, where the mean square error of the joint angle was less than 5°. Xiong et al. (2020) used random forest (RF) to establish a regression model based on sEMG to fit the angles of the knee and ankle joints during walking. The mean square errors of the knee and ankle joints were 6.64° and 3.89°, respectively.

While the aforementioned studies yielded satisfactory results, the practical application of lower-limb rehabilitation exoskeleton robots is intended for patients with motor dysfunction. Some patients may exhibit poor or absent EMG signals, thus necessitating the exploration of brain-computer interface technology as a potential solution (Vaughan et al., 2003; Vallabhaneni et al., 2005). Current brain signal acquisition techniques include electroencephalogram (EEG), functional magnetic resonance imaging (fMRI), and functional near-infrared spectroscopy (fNIRS). Among which fNIRS has the advantages of low cost, portability, and resistance to motion artifacts compared to other techniques (Krampe et al., 2018) and is more suitable for application scenarios with larger amplitudes, such as walking. Zhu et al. (2017) recognized the walking intention of three different step sizes based on fNIRS and obtained 83.3 % recognition accuracy. Jin et al. (2018) identified three different gaits based on fNIRS, namely, low speed in small step, medium speed in small step and low speed in medium step, with a classification accuracy of 78.79%. Nevertheless, the identified objects in the aforementioned studies correspond to discrete motion states, while the control of rehabilitation training equipment, such as exoskeletons, necessitates continuous motion parameters. Currently, there is a dearth of related research based on cerebral blood oxygen information.

In conclusion, this paper proposes a method that utilizes cerebral hemoglobin to fit human gait parameters, offering a novel solution for controlling rehabilitation exoskeletons in patients where effective collection of surface EMG signals from the lower limb is not feasible. Meanwhile, based on the cross-validation model, the individual adaptive enhancement of the cross-validation model was realized by the adaptive enhancement strategy only for a small amount of data of new individuals, and the gait parameter fitting accuracy was further improved.

## 2. Materials and methods

### 2.1. Participants

The experiment recruited 38 volunteers from Soochow University, aged 19–22 years, including 24 males and 14 females, with a male-to-female ratio of nearly 2:1. They were all involved in this walking experiment for the first time, were in good physical condition, and had no other adverse physical disease or psychiatric history. All volunteers volunteered to participate in the walking experiment and signed an informed consent form for the brain-computer interface, which was approved by the Ethics Committee of Soochow University.

### 2.2. Procedures and data acquisition

**FIGURE 1:**
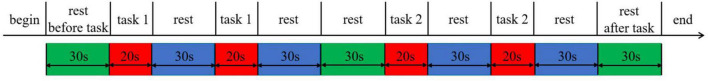
Experimental process. All participants were randomly divided into two groups. For the first group of participants, Task 1 represented walking with adjusted gait velocity, while Task 2 represented walking with adjusted stride length. The second group of participants had the opposite arrangement, where Task 1 represented walking with adjusted stride length, and Task 2 represented walking with adjusted gait velocity.

The experiment was divided into two types: speed adjustment and step adjustment. To eliminate the influence of objective factors on the experimental sequence, all subjects were randomly divided into two groups. The first group of participants initially maintained a constant stride length while gradually increasing their walking speed from low to high throughout the entire course. Subsequently, they maintained a constant walking speed while progressively increasing their stride length from small to large across the entire course. The second group followed the opposite sequence. Each type of walking experiment was repeated twice. Each subject performed a total of four walking experiments, and each completed walking experiment entered a rest state with a rest time of at least 30 s. The experimental process is illustrated in Figure 1.

**FIGURE 2:**
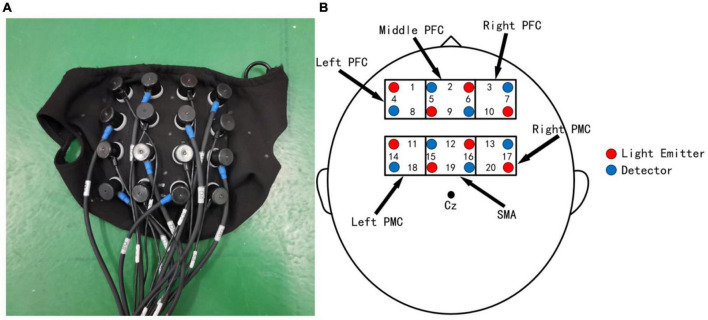
Layout of the headgear used in the experiments. **(A)** The head cap used for collecting cerebral blood oxygenation signals. **(B)** Probe layout and measurement of brain regions. The numbers represent channel numbers.

In the experiment, near-infrared brain imaging equipment (NirSmart) was applied to acquire brain hemoglobin information (Bu et al., 2020). Related studies (Fukuyama et al., 1997; Suzuki et al., 2004; Churchland et al., 2006; Harada et al., 2009; Holtzer et al., 2011, 2015; Caliandro et al., 2015) have shown that the cerebral cortex of prefrontal cortex (PFC), supplementary motor area (SMA), and primary motor cortex (PMC) are significantly associated with walking activity and play a key role in the adjustment of walking speed and step. Therefore, we focus on the changes in hemoglobin activity in these three brain regions with a 4*4 headgear layout (Figure 2). The inertial sensors (Xsens) were also applied to collect gait parameters, and according to the literature (McGrath et al., 2018), the knee joint was selected as the location for collecting motion information during the motion experiment.

### 2.3. Data analysis

To ensure the reliability and validity of brain hemoglobin data, it is crucial to eliminate the baseline drift from the brain hemoglobin signal (Okamoto et al., 2004). To remove baseline drift in the channel, subtract the value of the first sampling point from each data point in the channel’s samples. This adjustment shifts the oxygenation data to a position around zero. The heart rate and respiratory activities of the human body exhibit significant differences between walking and resting states, while the neuronal activity of the brain represents a focal research frequency band in BCI technology. The second-order Chebyshev filter is applied to band-pass filter the brain hemoglobin signal in five frequency bands (Stefanovska et al., 1999). They were heart rate effect (0.6∼2.0 Hz), respiration effect (0.145∼0.6 Hz), myogenic effect (0.052∼0.145 Hz), neurological effect (0.021∼0.052 Hz), endothelial cell metabolic activity (0.0095∼0.021 Hz). The gait parameters were subjected to a second-order Butterworth low-pass filter with a cutoff frequency of 6 Hz. Additionally, an absolute value operation was performed on the gait parameters, followed by smoothing using a moving average method. Additionally, while the research focuses on fitting gait parameters, it is important to note that these parameters dynamically change during each gait cycle, and the rotation angle and speed of each joint in the lower limbs during normal walking are primarily regulated by the lumbar nerve, rather than conscious control by the brain (Blumenfeld, 2002). Consequently, the research relies on the maximum speed/step (rotation angular velocity and rotation angle) within a gait cycle for digital step processing. This approach simplifies the fitting of gait parameters while also enabling the derivation of real-time gait parameter data for each gait cycle by considering the maximum speed/step parameters in conjunction with the statistical patterns of gait parameters within each cycle.

The temporal and spatial characteristics of 3 kinds of cerebral blood oxygen [oxygenated hemoglobin concentration (Hbo), deoxygenated hemoglobin concentration (Hbr), total hemoglobin concentration (Hbt)] in 5 frequency bands were extracted. Time domain features include energy, mean value, standard deviation, peak value, range, kurtosis, skewness, number of zero crossing and information entropy. The spatial domain features are calculated using Pearson correlation coefficients. Given the substantial number of sample points and features involved in a study, modeling time can be significantly prolonged. Moreover, the inclusion of redundant features may adversely affect the predictive performance of the model. Hence, it is imperative to employ correlation analysis and calculate feature importance scores to identify the most influential features for model fitting. This approach mitigates modeling time while enhancing predictive performance. Initially, the filtering method is employed to analyze the correlation coefficient (p) between each feature and gait parameters. A higher value of p suggests that the feature is more suitable for fitting gait parameters. Setting a threshold at 0.3, features with p greater than 0.3 are retained. Subsequently, the embedded method is employed to train the gradient boosting tree, enabling the acquisition of coefficients (c) for each feature. If the absolute value of the c is larger, it indicates that the feature has a greater contribution to fitting gait parameters. Finally, calculate the final score of the feature *f*_*score*_ (Formula 1):


(1) $$f_{s\,c\,o\,r\,e}=\frac{2\,p\,\left|c\right|}{p+\left|c\right|}$$

The features with larger *f*_*score*_ values are selected as the new feature space, and the AutoEncoder-Decoder framework (Hinton and Salakhutdinov, 2006) is employed for feature dimensionality reduction. This approach retains the key feature information while enhancing computational efficiency. The training set is divided into a cross-validation set and a test set, and the model is trained using 4-fold cross-validation. Prior to model training, the feature data and inertial sensor data are Z-Score normalized to reduce individual variability. The long short term memory (LSTM) model (Sherstinsky, 2020) was applied to establish a regression model for fitting the gait parameters, and L2 regularization was used to prevent overfitting during model training. An incremental learning approach is utilized for adaptive training, building upon a small amount of volunteer data, utilizing the LSTM model as the foundation. Zhang et al. (2014) conducted a study on decoding continuous upper limb movements based on EMG signals. They employed an adaptive training approach to decode continuous upper limb movements from EMG signals. Compared to non-adaptive methods, the training results were more accurate. During the adaptive training process, the coefficient of R2 was used as the evaluation criterion. If the R2 value improved, the new model parameters were retained; otherwise, the original model parameters were kept.

The primary evaluation indicators for model fitting include the relative root mean square error (RMSE, Formula 2) and the coefficient of determination (R2, Formula 3).


(2) $$R\,M\,S\,E=\sqrt{\frac{\sum_{i=1}^{m}{\left(\frac{y_{i\,P\,r\,e\,d}-y_{i\,R\,e\,a\,l}}{y_{i\,R\,e\,a\,l}}\right)}^{2}}{m}}$$

(3) $$R\,2=1-\frac{\sum_{i=1}^{m}{\left(y_{i\,P\,r\,e\,d}-y_{i\,R\,e\,a\,l}\right)}^{2}}{\sum_{i=1}^{m}{\left(y_{i\,R\,e\,a\,l}-y_{M\,R\,e\,a\,l}\right)}^{2}}$$

where *y*_*iPred*_ represents the ith sampling point corresponding to the model-fitted gait parameter, *y*_*iReal*_ represents the ith sampling point corresponding to the true gait parameter, and *y*_*MReal*_ represents the average of all sampling points corresponding to the true gait parameter.

RMSE measures the average difference between predicted values and actual values. A lower RMSE indicates a better fit, as it suggests that the predicted values are closer to the actual values. R2 represents the proportion of variance in the dependent variable that can be explained by the independent variables. It ranges from 0 to 1, where 1 signifies a perfect fit and 0 indicates no relationship between the variables. When fitting gait parameters, a lower RMSE and a higher R2 indicate higher model accuracy and improved fit.

## 3. Results

### 3.1. Extraction and analysis of features

Initially, the cerebral hemoglobin signal is processed to remove baseline drift and band-pass filtered in five frequency bands by the second-order Chebyshev filter. The gait parameters are processed by absolute value after low-pass filtering, then smoothed by moving average method, and finally processed by ladder line in the adjacent minimum range. Features were extracted from the pre-processed brain hemoglobin signal, and the features were calculated from the perspective of time domain and spatial domain, respectively. These features are 5 (1–5 frequency band) * 3 (brain hemoglobin HbO, HbR, HbT) * (6*9 + 15) (temporal and spatial domain characteristics) = 1,035.

**FIGURE 3:**
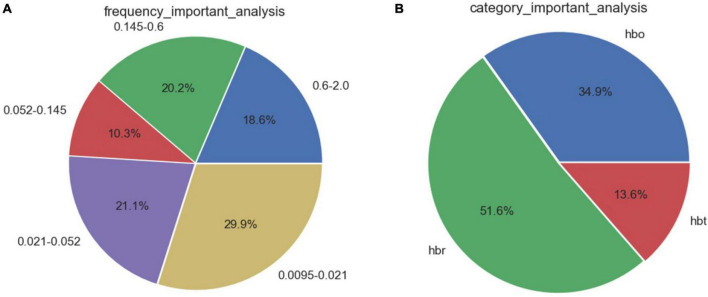
Feature importance distribution corresponding to the speed parameters. **(A)** Analysis of feature importance across different frequency bands. **(B)** Analysis of feature importance for different types of cerebral oxygenation.

**FIGURE 4:**
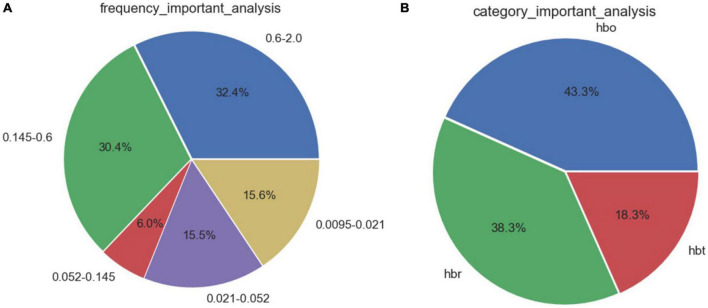
Feature importance distribution corresponding to step length parameters. **(A)** Analysis of feature importance across different frequency bands. **(B)** Analysis of feature importance for different types of cerebral oxygenation.

Feature selection based on correlation analysis and feature importance score. For speed parameters (Figure 3), the frequency bands of neural activity and endothelial cell metabolic activity accounted for 50.0% of the 5 frequency bands. The proportion of deoxyhemoglobin in the three types of blood oxygen was as high as 51.6%. For the step length parameter (Figure 4), the heart rate and respiratory activity frequency band accounted for 62.8% of the 5 frequency bands. The proportion of oxyhemoglobin in the three types of blood oxygen was as high as 43.3%.

### 3.2. Gait parameter adaptive fitting results

A total of thirty subjects were selected from the experimental sample and divided into training and testing sets in an 8:2 ratio. The model was trained using 4-fold cross-validation. During the training of the model, a forgetting rate of 0.2 was set, along with 200 iterations. Additionally, an early stopping mechanism based on minimizing RMSE was incorporated. During the 200 iterations of the training process, if the RMSE value does not decrease for eight consecutive iterations, the model stops training.

The final statistical results of the fitted gait parameters for the 30 volunteers showed that the RMSE of the cerebral blood oxygen information for the fit of the gait speed parameters was 1.361% and the R2 was 71.554%, the RMSE for the fit of the gait length parameters was 3.321% and the R2 was 72.022%.

**FIGURE 5:**
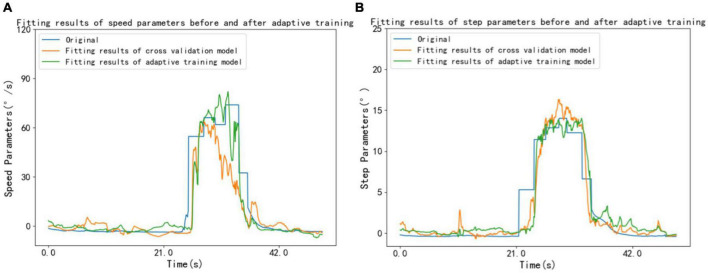
A volunteer’s speed and step parameters before and after adaptive training. **(A)** Speed parameters **(B)** step parameters.

The LSTM model was adaptively trained using 8 unused subject data from a pool of 38 volunteers. Following adaptive training, there was an average decrease of 0.303% in the RMSE of individual speed parameters, accompanied by an average increase of 9.851% in R2. Similarly, there was an average decrease of 1.198% in the RMSE of individual step parameters, accompanied by an average increase of 6.985% in R2. Figure 5 shows the speed parameters and step parameters of a volunteer before and after adaptive training.

## 4. Discussion

This study proposes a method for fitting human continuous gait parameters based on cerebral hemoglobin. At present, physical sensors and EMG information are mainly used to identify gait parameters in the control of the lower extremity exoskeleton. However, some patients have poor-quality of EMG signals or no signals, which may cause secondary injuries to patients. While some studies (Shen et al., 2022; Zhu et al., 2022) used EEG information to identify gait to achieve real-time control of the exoskeleton, the practical application of EEG signals is hindered by susceptibility to interference from complex external electromagnetic environments, leading to suboptimal recognition outcomes. The fNIRS devices possess characteristics such as portability and low sensitivity to the testing environment, making them more suitable for real-world scenarios in the clinical application of rehabilitation exoskeleton devices. This study focused on maintaining a continuous variation of gait parameters during the walking experiments, with specific values determined by the participants’ own height and walking habits. It was not required for each participant to achieve a specific absolute value of gait parameters during the walking experiments. The emphasis of this study was to stimulate the spontaneous nature of participants’ movements and to align the walking experiments with their real-life walking experiences. These are conducive to the application of rehabilitation exoskeleton equipment.

Most of the current studies based on cerebral blood oxygen are to distinguish between rest and motion states or to identify discrete motion states while fitting continuous motion parameters is still in the preliminary exploration stage. We established an LSTM regression model for fitting continuous gait parameters through brain hemoglobin information. The RMSE of the speed parameters fitting was 1.361%, and the R2 was 71.554%. The RMSE of the step parameters fitting is 3.321%, and the R2 was 72.022%. The results tentatively demonstrate the feasibility of use cerebral blood oxygen to fit gait parameters for exoskeleton control when the EMG signals of the lower limbs cannot be collected.

For the purpose of high-fitting model applicability, an individual adaptive optimization strategy based on the cross-validation model and individual added data is proposed. The cross-validation model is trained adaptively by incremental learning method to retain the optimal model parameters adapted to individuals. Following adaptive training, there was an average decrease of 0.303% in the RMSE of individual speed parameters, accompanied by an average increase of 9.851% in R2. Similarly, there was an average decrease of 1.198% in the RMSE of individual step parameters, accompanied by an average increase of 6.985% in R2. The cross-validation model can be directly applied to all participants without individual training and learning. The adaptive parameter learning based on the cross-validation model can quickly optimize the fitting effect of gait parameters according to the new data of individuals and improve the adaptiveness of the model output, which is helpful to increase the practicality in fitting individual joint motion parameters scenarios and reduce the discomfort caused by misjudgment due to the difference of individual walking habits.

Furthermore, this study has certain limitations. The experiment recruited 38 subjects, all aged between 19 and 22 years, resulting in a relatively young age distribution. The practical application object of the lower limb rehabilitation exoskeleton robot should be the public. Belli et al. (2021) found differences in cerebral oxygenation activity in the cortical regions of the brain during walking between young and elderly individuals. Therefore, future research should aim to collect brain oxygenation data from individuals across different age groups to validate the applicability of this study’s findings. Additionally, there is a need to establish an online validation platform to assess the feasibility of using brain oxygenation information to control exoskeleton devices. This would allow the research results to be effectively applied in the rehabilitation of individuals with movement impairments.

Currently, this paper provides only a preliminary exploration of the feasibility of fitting gait parameters using cerebral blood oxygen, leaving ample room for enhancing the accuracy of the fitted model. As the study is based on the maximum value of a gait cycle (speed/step) for the step process, the gait parameters are also fitted to characterize the maximum value of the current cycle. Therefore, the parameters fitted in a gait cycle should theoretically be the same and should not change significantly in the short term (e.g., 0.5 s) after a significant change in gait parameters. In future research, efforts will be made to optimize this issue and minimize the occurrence of false positives within a single gait cycle. At the same time, this study has not been compared with other filtering methods or fitting algorithms, so more filtering algorithms and fitting algorithms need to be tried in the future to improve the accuracy of the model.

## 5. Conclusion

This study established an LSTM regression model for fitting continuous gait parameters through brain hemoglobin information. For the fitting of speed parameters, the RMSE was 1.361% with an R2 of 71.554%. Meanwhile, the fitting of step parameters resulted in an RMSE of 3.321% with an R2 of 72.022%. Additionally, incremental learning of the cross-validation model was performed using individual volunteer data. The RMSE of all individual speed parameters decrease on average 0.303%; R2 average increase 9.851%; The RMSE of all individual step parameters decrease on average 1.198%; R2 average increase 6.985%. The research results preliminarily prove the feasibility of fitting continuous motion parameters based on cerebral blood oxygen information. This scheme can provide a new rehabilitation exoskeleton control scheme for patients who cannot collect effective lower limb EMG, improve the applicability of rehabilitation exoskeleton equipment, and lay a certain foundation for the full application of BCI technology in the field of sports rehabilitation.

## Data availability statement

The raw data supporting the conclusions of this article will be made available by the authors, without undue reservation.

## Ethics statement

The studies involving human participants were reviewed and approved by the Ethics Committee of Soochow University. The patients/participants provided their written informed consent to participate in this study.

## Author contributions

YZ, CL, and HM conceived and designed this study. YZ and YP collected experimental data. HM and YZ conducted literature search, data analysis, and first draft writing. CL and LS provided research support and were responsible for supervision. HM and CL completed the editing of the final manuscript. All authors read and approved the final manuscript.
